# See your mental state from your walk: Recognizing anxiety and depression through Kinect-recorded gait data

**DOI:** 10.1371/journal.pone.0216591

**Published:** 2019-05-22

**Authors:** Nan Zhao, Zhan Zhang, Yameng Wang, Jingying Wang, Baobin Li, Tingshao Zhu, Yuanyuan Xiang

**Affiliations:** 1 CAS Key Laboratory of Behavioral Science, Institute of Psychology, Chinese Academy of Sciences, Beijing, China; 2 School of Computer Science and Technology, University of Chinese Academy of Sciences, Beijing, China; Jinling Clinical Medical College of Nanjing Medical University, CHINA

## Abstract

As the challenge of mental health problems such as anxiety and depression increasing today, more convenient, objective, real-time assessing techniques of mental state are in need. The Microsoft Kinect camera is a possible option for contactlessly capturing human gait, which could reflect the walkers’ mental state. So we tried to propose a novel method for monitoring individual’s anxiety and depression based on the Kinect-recorded gait pattern. In this study, after finishing the 7-item Generalized Anxiety Disorder Scale (GAD-7) and the 9-item Patient Health Questionnaire (PHQ-9), 179 participants were required to walked on the footpath naturally while shot by the Kinect cameras. Fast Fourier Transforms (FFT) were conducted to extract features from the Kinect-captured gait data after preprocessing, and different machine learning algorithms were used to train the regression models recognizing anxiety and depression levels, and the classification models detecting the cases with specific depressive symptoms. The predictive accuracies of the regression models achieved medium to large level: The correlation coefficient between predicted and questionnaire scores reached 0.51 on anxiety (by epsilon-Support Vector Regression, e-SVR) and 0.51 on depression (by Gaussian Processes, GP). The predictive accuracies could be even higher, 0.74 on anxiety (by GP) and 0.64 on depression (by GP), while training and testing the models on the female sample. The classification models also showed effectiveness on detecting the cases with some symptoms. These results demonstrate the possibility to recognize individual’s questionnaire measured anxiety/depression levels and some depressive symptoms based on Kinect-recorded gait data through machine learning method. This approach shows the potential to develop non-intrusive, low-cost methods for monitoring individuals’ mental health in real time.

## Introduction

Anxiety disorders and depression were the two most common mental disorders [1], which brought great challenge to personal wellbeing and social economy around the world [2]. The population with anxiety and depression had grown from 416 million in 1990 to 615 million in 2013 [2], and these two problems accounted 55.1% of the global burden of mental disease in terms of disability-adjusted life years [3]. The internet-based and mobile-based interventions have become recognized as an important means of dealing with this challenge and improving psychological wellbeing in large populations [4, 5]. Based on this trend, the need of more convenient, objective, real-time assessing of user’s mental state appears to be more and more urgent.

As the most commonly used tools, a series of questionnaires capturing anxious or depressive symptoms had been developed and showed great success in psychiatric practice. However, purely relying on self-report questionnaires also limited the availability and effectiveness of today’s mental health service. First, filling out a questionnaire could often be an extra burden for users and sometimes the quality of the answers is hard to be guaranteed in practice [6]. Second, it is usually not feasible to require the users to answer the same questions repeatedly and frequently, which could make the questionnaire not suitable for a real-time assessing of the dynamic nature of mental states.

As a natural, easily observed body movements, human gait has been found to reflect walker’s mental aspects, including the state of anxiety and depression. As the typical symptoms of anxiety disorders, fear could be recognized by human observers through the head upright and elbows bent during walking [7] or the posture with backward head bend, forearms raised and weight shift backward [8]; anxious state could be reflected in low energy, slow movement and expanded limbs and torso torso [9]. The electro-myographic (EMG) study also found increased muscle tension in patients with generalized anxiety disorder (GAD), which would affect their body movements [10]. As the typical symptom of depression, sadness during walking were reflected by reduced walking speed, reduced arm swing, reduced vertical head movements, larger lateral swaying movements of the upper body and a more slumped posture [11]. Hand movements and head-down position in walking were found to be different in neutral and depressed participants [12], and depressed patients showed significantly lower gait velocity, reduced stride length, increased double limb support [13] and larger swing time variability [14].

Although there has been much evidence that anxiety and depression could be reflected in gaits, which could be an objective, easily accessible data source, the methods of gait-based mental health state detection has not yet been fully established. The previous findings only presented some gait features statistically different between target and control groups (e.g., [7, 11]), but did not provide any comprehensive model to recognize the status of anxiety or depression using those features. There is no evidence that the gait features found in the anxiety/depression group were specific for anxiety/depression, so till now we cannot make any assessment on anxiety/depression just based on single or a couple of those gait features. Besides, a practical method should also include convenient tools to record gaits data. The need of expensive, complex facilities (e.g., the Motion Capture System) or human perceivers in previous studies made them not much of a basis for a real-life solution.

The use of Kinect may be a practical option to conveniently record gaits data in real life. With the advantage of portability and low cost, the marker-free sensor system Microsoft Kinect which is designed for Xbox [15, 16] could conduct a continuous monitoring of body movement patterns from a three-dimensional perspective. It has been demonstrated that Kinect has acceptable validity in estimating body posture and motion [17–19]. In the laboratory, with the use of Kinect researchers were able to distinguish different daily human activities [20], identify the gait cycles in treadmill [21] and record the trace of people’s simple step movements [22]. And this capability of monitoring body movements was soon used in clinical applications [23, 24]. As Hondori and Khademi [25] reviewed, Kinect could bring certain benefits as a part of rehabilitation system for the patients of stroke, Parkinson’s, cerebral palsy and some other neurological disorders, and have the potential to be a reliable solution for telerehabilitation [26]. Further more, Li et al. [27] tried to detect induced emotions from Kinect-recorded gait data, showing that through Kinect, researchers could not only analyze the movement itself, but also possibly recognize some motion-reflected mental states.

To establish a anxiety/depression detection method based on natural gaits using Kinect, we need to build computational models which could recognize anxiety/depression based on Kinect-recorded gaits data, rather than only find some gait features relevant to anxiety or depression. In order to reach this goal, we would try to extract low-level features from configurations directly described by values of 3D coordinate, and construct computational models using machine learning methods to automatically recognize the levels of anxiety and depression. These data-driven low-level features could not provide a high-level description of the gaits pattern of anxiety or depression, such as walking speed, arm swing, head movements, etc., but may carry more complete information which would be utilized by the computational models to detect anxiety or depression. This approach has been shown feasible in the field of affective computing [28, 29].

In the present study, we hypothesized that the questionnaire measured anxiety and depression levels could be recognized based on individual’s natural gaits, and the computational model could be built through machine learning methods using Kinect-recorded data. We conducted an experiment to test this hypothesis.

## Materials and methods

### Participants and apparatus

In this study, we recruited 179 graduate students (100 males, 79 females) with an average age of 24.2(SD = 1.5) from the University of Chinese Academy of Sciences. All the participants enrolled in this experiment reported no physical disease or injury which affects daily walking. The experiment environment was set to similar as the one in Li et al.’s study [27], including a 6*m* * 1*m* footpath with two Kinect 2.0 cameras placed at the beginning and the end of the footpath.

### Data collection procedures

After signing an institutionally approved informed consent, each participant was firstly required to complete a series of questionnaires. Besides basic demographic information, the questionnaires included the 7-item Generalized Anxiety Disorder Scale (GAD-7) [30], which asks about the states in past two weeks to calculate an anxiety score, and the 9-item Patient Health Questionnaire-Depression (PHQ-9) [31], which asks about the depressive symptoms in past two weeks to calculate a depression score. These two questionnaires are widely used as screening tools for assessing and monitoring anxiety and depression severity, to assist the clinician in making diagnosis. Both of them showed excellent internal reliability (.92 for GAD-7; .86-.89 for PHQ-9) and test-retest reliability (.83 for GAD-7; .84 for PHQ-9) [30, 31]. The good sensitivity and specificity of GAD-7 for detecting anxiety disorders and of PHQ-9 for detecting depressive disorders had also been proved by many previous studies, with the usual cutpoint ≥10 for both the two scales [32]. All the participants finished GAD-7, while 167 valid samples with PHQ-9 scores were achieved (95 males, 72 females; Mean Age = 24.2, SD = 1.5).

Secondly, all the participants were asked to walk on the footpath, back and forth naturally as their daily performance, for two minutes with Kinect cameras continuously shooting in order to make sure of adequate high-quality gait record. The protocol had obtained permission from the Institutional Review Board of the Institute of Psychology, Chinese Academy of Sciences. (approved number: H15010).

### Data preprocessing

#### Denoise

The original record from Kinect was the 3-dimentinal accelerations of the 25 main body joints [27], by 30*Hz* sampling rate. The Sliding Window Gaussian Filtering [33] was firstly conducted on the original data of each body joint to remove the noise and smooth the records. With the window size as 5 and the convolution kernel *c* = [1, 4, 6, 4, 1]/16, the denoising process is defined as:

$$Out\left[i\right]=\frac{1}{16}\left(In\left[i\right]\times 1+In\left[i+1\right]\times 4+In\left[i+2\right]\times 6+In\left[i+3\right]\times 4+In\left[i+4\right]\times 1\right).$$ (1)

**Fig 1:**
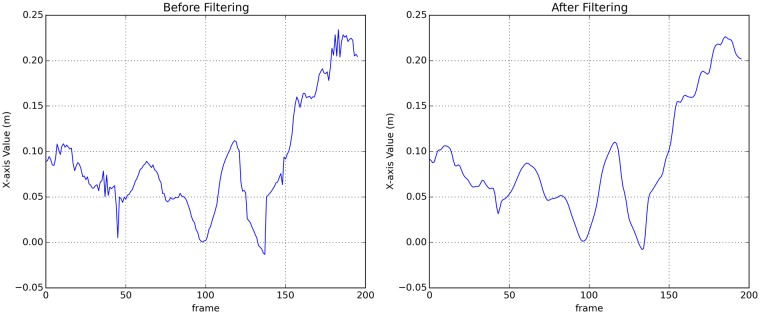
The comparison of recorded data before and after Gaussian filtering. A: Before filtering. B: After filtering.

*In* refers to the original time series data recorded by Kinect, and *Out* refers to the smoothed time series data. Fig 1 shows a segment of one single joint’s X-axis data before and after denoise. The time series data processed by Gaussian Filter (Fig 1A) is obviously smoother than the original data (Fig 1B).

#### Coordinate system transformation

Using the Kinect-default 3D coordinates with the camera position as the origin may cause considerable mistake in the process of gait pattern analysis, due to the different positions relative to Kinect camera of different participants during walking. As a solution, in each frame (containing 25 main body joints) we replaced the coordinate system with the position of SpineBase joint as the origin point, and the data of the rest 24 joints would be used in next steps.

#### Resampling

As a non-intrusive recording method, shortening the necessary recording time would increase its practical value, so we tried to select shorter time series from each participant during resampling. We divided the two minutes long recording of each Kinect into front and back segments, based on whether the participant were facing to the camera. Since the accuracy of joints tracking was better while the participant facing to the camera, the back segments and the frames of turning were dropped.

Since walking is a periodical body movement, the final data we used should cover at least one cycle. Meanwhile, our feature extraction method Fast Fourier Transform required a data length equal to power of 2. So we chose 64 frames (about 2s) as the length of the final data segments used in feature extraction. We firstly cut the front segments into several 64-frame long small segments, each of which was perfectly continuous without any invalid frame. Then we randomly selected one 64-frame segment from each participant, as the final sample used in feature extraction.

### Feature extraction

For feature extraction, Fast Fourier Transforms (FFT) [34] (defined as Eq (2)) were conducted. *N* refers to the length of the data segment, and *x*_*n*_ (*x* ∈ {*X*, *Y*, *Z*}) refers to the preprocessed gait data. We calculated the amplitude of FFT *X*_*k*_ which converts the sampled function from its original domain (time domain) to the frequency domain for each joint axis (X, Y and Z), and got 64 amplitude coefficients from each axis as features. Then we used the Z-score function to normalize these features.

$$X_{k}=\sum_{n=0}^{N-1}x_{n}e^{-i2\pi k\frac{n}{N}}\;k=0,\cdots ,N-1,$$ (2)

### Feature selection

To minimize the error caused by redundant information and improve predictive accuracy, we conducted the Pearson correlation, one of the most commonly used methods for feature selection [35, 36]. The correlation coefficients were calculated between anxiety/depression score and each feature (FFT amplitude) on each axis. Then, on each axis, we selected the 5 features with the largest absolute value of correlation coefficients, generating a total of 360 selected features (5 * 3 * 24 = 360) for each participant.

### Model training

To predict the anxiety and depression scores, we trained models using five frequently used regression algorithms, i.e. Simple Linear Regression (SLR), Linear Regression(LR), epsilon-SVR (e-SVR), nu-SVR (n-SVR) and Gaussian Processes (GP), and applied 10-fold cross validation to test each model, which means that we randomly selected 10% of the sample for testing and used the rest of the sample for training, and repeated this process ten times for each model. The Pearson correlation coefficient between the predicted scores of each model and the questionnaire scores was calculated as the predictive accuracy index of each model.

As each of the PHQ-9 items represents a unique symptom of depression in DSM-IV criteria, the score of each item is also helpful to assess the specific symptom of an individual besides the overall score [31]. For each item, the scale ranges from 0 (not at all) to 3(nearly every day), and we divided our samples into the symptomatic group (scoring 1-3) and non-symptomatic group (scoring 0). Then we tried to build classification models on each item to find out the cases with that symptom. We utilized the algorithms of Simple Logistic (SL), K-Star (K-S) and C-SVC (C-SVC), and tested the models through 10-fold cross validation. The precision, recall and F-measure were calculated as the measurement of the predictive accuracy, which are commonly used to evaluate classification models in machine learning: Precision is the fraction of the cases with symptom among the cases retrieved by the model; recall is the fraction of the model-retrieved symptomatic cases among all the cases with symptom; and F-measure is the harmonic mean of both precision and recall.

The model training and testing process was conducted through WEKA3.8, a tool as the collection of machine learning algorithms for data mining tasks.

## Results

### Questionnaire scores of anxiety and depression

**Fig 2:**
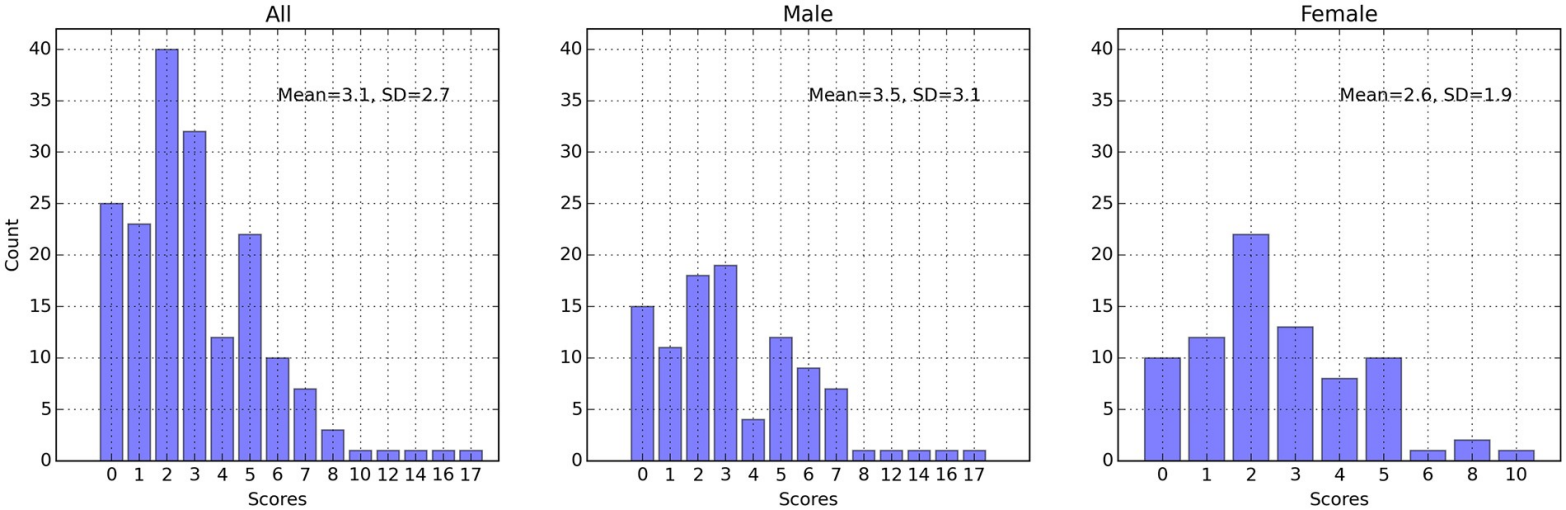
The distributions of the whole sample, males, and females on the GAD-7 scores. A: All. B: Male. C: Female.

**Fig 3:**
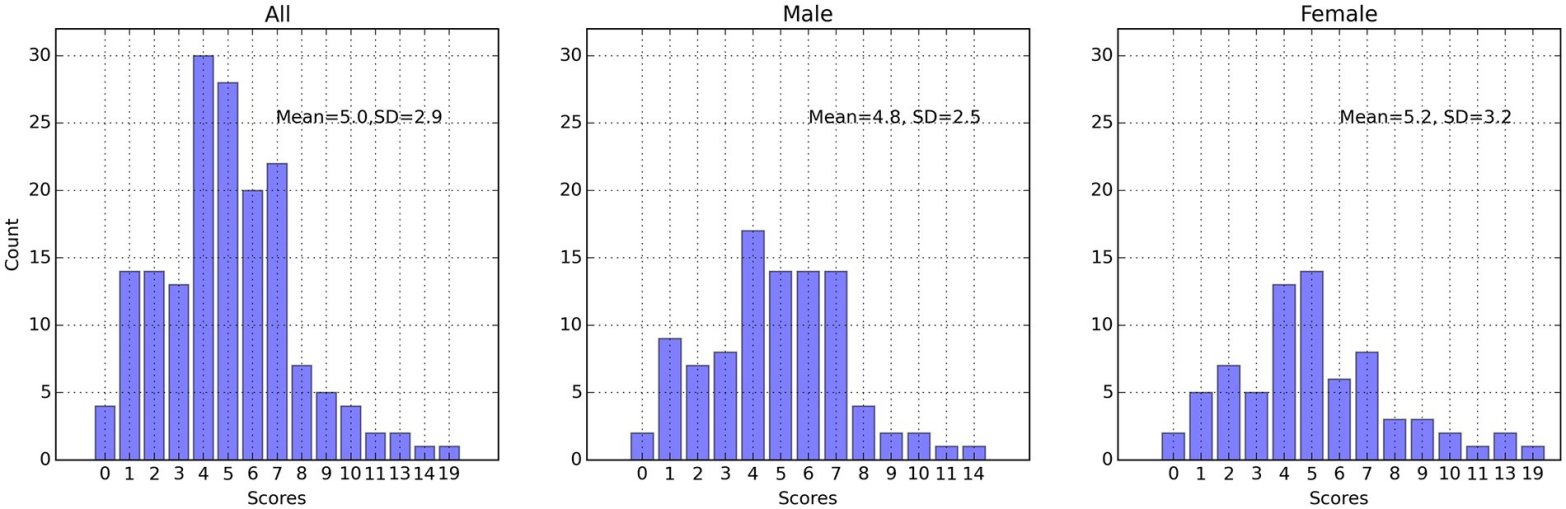
The distributions of the whole sample, males, and females on the PHQ-9 scores. A: All. B: Male. C: Female.

The distributions of questionnaire scores of anxiety and depression were shown in Figs 2 and 3. In our sample males showed higher anxiety scores than females (p = .039, df = 177), while the depression scores between two genders showed no significant difference (p = .442, df = 165). The anxiety and depression scores of both genders generally distributed in the relatively healthy region, while a few cases had anxious or depressive symptoms of different severities. Since we would also build the models to recognize each symptom in PHQ-9, the sampling distribution on each item of PHQ-9 were also presented in Table 1.

**Table 1:** The sampling distribution on the PHQ-9 items (the whole sample).

	0: Not at all	1: Several days	2: More than half the days	3: Nearly every day
Item 1. Losing interest or pleasure.	25	136	4	1
Item 2. Feeling down or depressed.	57	107	3	0
Item 3. Sleep problems.	78	73	11	5
Item 4. Low energy.	41	115	11	0
Item 5. Eating problems.	85	67	11	4
Item 6. Feeling of failure.	103	60	3	1
Item 7. Trouble in concentration.	74	75	13	5
Item 8. Moving or speaking too slow or being fidgety.	134	30	2	1
Item 9. Thoughts of suicide.	163	4	0	0

Note: table entries are the numbers of cases.

### The recognition of anxiety levels by regression models

The predictive accuracies of the regression models on GAD-7 score were presented in Table 2. The performances of different models varied considerably. While training and testing the models using the whole sample, the correlation coefficients between predicted and questionnaire scores achieved 0.51 (by e-SVR). If building models separately on males and females, the predictive accuracies could be even higher, up to 0.61 (on males by GP) and 0.74 (on females by GP).

**Table 2:** Predictive accuracies of the regression models on GAD-7 scores.

	SLR	LR	e-SVR	n-SVR	GP
*All*	-0.07	0.24**	**0.51** ***	0.48***	0.43***
*Males*	0.01	0.54***	0.56***	0.53***	**0.61** ***
*Females*	0.29*	0.69***	0.62***	0.56***	**0.74** ***

Note: table entries are Pearson correlation coefficients (r).

* *p*<.05

** *p*<.01

*** *p*<.001

### The recognition of depression levels by regression models

The predictive accuracies of the regression models on PHQ-9 score were presented in Table 3. The performances of different models also varied. While using the whole sample, the correlation coefficients between predicted and questionnaire scores achieved 0.51 (by GP). And if the model being trained separately by data from different genders, the predictive accuracies also changed. For males it was 0.45 (by GP), and for females it was up to 0.64 (by GP).

**Table 3:** Predictive accuracies of the regression models on PHQ-9 scores.

	SLR	LR	e-SVR	n-SVR	GP
*All*	-0.16*	0.23**	0.38***	0.40***	**0.51** ***
*Males*	0.20	0.32**	0.24*	0.30**	**0.45** ***
*Females*	0.05	0.60***	0.41***	0.43***	**0.64** ***

Note: table entries are Pearson correlation coefficients (r).

* *p*<.05

** *p*<.01

*** *p*<.001

### The detection of cases with different depressive symptoms by classification models

In Table 4, the precision, recall and F-measure of the three classification models on each symptom are presented. For some symptoms, such as Item 1, Item 2 and Item 4, the predictive accuracies were relatively high. The recall on these items achieved over 0.9 while the precision could be around 0.7 or higher, which means that the models could help us to find out more than 90% cases with these symptoms, with less than 30% false alarms. On some other symptoms, such as Item 3, Item 5, Item 6 and Item 7, our models also showed some effectiveness, especially for the models of LR and C-SVR, and their predictive accuracies varied in the whole sample, males and females. The predictive accuracies on Item 8 were low, and for Item 9, the symptomatic cases were too few to train the models.

**Table 4:** Predictive results of the classification models on each symptom in PHQ-9.

		ALL			Male			Famele		
		LR	K-S	C-SVC	LR	K-S	C-SVC	LR	K-S	C-SVC
**Item 1**	P	**0.85**	**0.85**	**0.84**	**0.87**	**0.87**	**0.91**	**0.83**	**0.83**	**0.84**
	R	**0.95**	**0.99**	**0.94**	**0.97**	**0.98**	**0.92**	**0.95**	**0.96**	**0.80**
	F	**0.90**	**0.92**	**0.89**	**0.92**	**0.93**	**0.91**	**0.89**	**0.89**	**0.82**
**Item 2**	P	**0.67**	**0.66**	**0.66**	**0.61**	**0.60**	**0.61**	**0.67**	**0.70**	**0.70**
	R	**0.86**	**0.95**	**0.98**	**0.91**	**0.93**	**0.96**	**0.80**	**0.94**	**0.99**
	F	**0.76**	**0.78**	**0.79**	**0.73**	**0.73**	**0.74**	**0.73**	**0.80**	**0.82**
**Item 3**	P	0.59	0.56	**0.64**	0.58	0.60	**0.62**	**0.65**	0.56	**0.74**
	R	0.68	0.91	**0.69**	0.54	0.15	**0.74**	**0.62**	0.92	**0.64**
	F	0.66	0.69	**0.66**	0.56	0.25	**0.67**	**0.63**	0.70	**0.69**
**Item 4**	P	**0.78**	**0.76**	**0.76**	**0.73**	**0.70**	**0.75**	**0.82**	**0.79**	**0.85**
	R	**0.96**	**0.96**	**0.99**	**0.94**	**0.98**	**0.77**	**0.93**	**0.95**	**0.91**
	F	**0.86**	**0.85**	**0.86**	**0.82**	**0.82**	**0.76**	**0.88**	**0.86**	**0.88**
**Item 5**	P	**0.66**	0.49	0.62	0.58	0.47	**0.66**	**0.66**	0.49	**0.63**
	R	**0.61**	0.89	0.65	0.53	0.88	**0.68**	**0.69**	0.92	**0.60**
	F	**0.63**	0.64	0.63	0.55	0.61	**0.67**	**0.67**	0.64	**0.62**
**Item 6**	P	**0.66**	0.57	0.75	0.19	0.43	0.42	0.40	0.53	0.60
	R	**0.61**	0.06	0.14	0.11	0.11	0.36	0.28	0.25	0.17
	F	**0.63**	0.11	0.24	0.14	0.17	0.39	0.32	0.34	0.26
**Item 7**	P	0.37	0.57	0.46	0.54	0.71	0.51	**0.61**	0.58	**0.63**
	R	0.20	0.88	0.50	0.51	0.13	0.64	**0.63**	0.91	**0.65**
	F	0.26	0.70	0.48	0.53	0.22	0.57	**0.62**	0.71	**0.64**
Item 8	P	0.33	0.25	0.31	-	0.33	0.2	0.50	1.00	0.24
	R	0.09	0.06	0.33	-	0.14	0.14	0.11	0.16	0.21
	F	0.14	0.10	0.32	-	0.2	0.2	0.17	0.27	0.22

Note: Due to the small sample size of item 9 symptomatic group, no valid results were obtained. The results are marked in bold if the precision and recall are both higher than 0.6.

P refers to precision;

R refers to recall;

F refers to F-measure.

## Discussion

Our results supported the hypothesis that the individual’s questionnaire measured severities of anxiety and depression could be recognized based on their natural gaits, with the predictive models built through machine learning. For both anxiety and depression, the correlation between predicted anxiety/depression scores and self-reported questionnaire scores achieved medium to large level (0.43 ∼ 0.74). Furthermore, the classification models were effective in detecting the cases with several depressive symptoms. These results indicated two facts. First, the individual’s anxiety and depression degrees did be reflected in the natural gaits, which is consistent with the previous studies revealing the gait features relevant to anxiety and depression (e.g., [7, 9, 11, 13]). Second, our results also showed that no matter to what extent this target information in gaits could be visually inspected, it could be measured and utilized in recognition with the help of electronic devices.

The effective predictive model in the current study was built through machine learning method, based on the low-level features directly extracted from the original 3*D* coordinates of the walker’s main body joints. The high-level feature descriptors of body movements in this field often appeared to be based on subjective, qualitative evaluations [37], which restricted the practice integrating different features into one predictive model. The low-level features in our study (FFT amplitudes) may not provide any intuitive understanding of individual’s gait, however, it could cover the information of target psychological aspects reflected in gaits more comprehensively. Our results showed the validity of the computing model based on the low-level features in recognizing questionnaire measured severities of anxiety and depression, and showed the potential of this data-driven approach in the field of psychometrics.

The apparent differences among the model effectiveness in detecting different depressive symptoms (Table 4) bring us more information about the usage of the predictive model. Considering both precision and recall, our classification models performed well in screening losing interest or pleasure, feeling down or depressed, and low energy. But for other symptoms like sleep problem, eating problem, feeling of failure and trouble in consideration, our models showed relatively lower effectiveness, or even no effect for recognizing moving or speaking too slow or being fidgety. Although these results may be affected by different distributions of item scores, they suggested that some of depressive symptoms are reflected in gaits more strongly than others. There are two scoring methods of PHQ-9 in clinical practice [38]: the cut-off based on summed-item scores, and the algorithm based on DSM-IV criteria, which requires a total of at least five symptoms rated as at least more than half the days except the suicidal ideation item, and also requires at least one of the first two symptoms of PHQ-9 (losing interest or pleasure; feeling down or depressed) scored as at least more than half the days. As the summed-item method is more sensitive and has been dominant in the screening of depression [38], the prediction of the total score of PHQ-9 has greater value in practice. Meanwhile, the detection of certain items provides additional information of the subject’s symptom appearance, but is still not able to support the algorithm scoring method, as it is not valid for all the items.

In our study the predictive accuracies of the models trained by different machine learning algorithms also showed great disparity, which may be seen as a clue suggesting the relationships between the features we used and anxiety/depression. SLR and LR were linear regression models, while e-SVR, n-SVR and GP were nonlinear regression models. The outstanding performances of nonlinear regression models in our study implied that the relationship between the gait information and anxiety/depression was more possible to be nonlinear rather than linear. It may be one reason of that the specificity of the gait patterns relevant to anxiety and depression in previous studies were not unambiguously permitted [11].

For both anxiety and depression scores, if we trained and tested regression models on males and females separately, the predictive accuracy on females was higher than on males. In detecting the symptomatic cases, models trained on females also performed better on some symptoms, such as feeling down or depressed and trouble in concentration. Intuitively, it seemed that for females the level of their anxiety and depression could be reflected in their gaits more obviously than for males, in other words, women seemed to “express” their anxiety and depression more through natural gaits than men, especially for some symproms. As many studies shown, there existed some difference on males’ and females’ symptoms of anxiety/depression (e.g., [39–41]). Researchers claimed that women may receive more positive reinforcement for expressing concerns toward anxiety symptoms [42], and men with depression showed impairment at lower symptoms levels than women [43], and reported consistently fewer symptoms than women [44]. These findings were consistent with the inference in our study that females’ gaits could reflect their anxiety/depression better than males. Although we have not seen any reports revealing gender difference of anxiety/depression symptoms in terms of gaits, it may be valuable to conduct such comparison in future study.

As a pilot study, it is appropriate to highlight several limitations. First, the current study used questionnaire-based scales of anxiety and depression symptoms but not a clinical diagnosis of either. Although the validity of the questionnaires as a screening tool in accessing anxiety and depression severity has been well proved in literatures [32], the questionnaire score itself cannot be used as diagnosis. Second, the sample in this study was composed of graduate students rather than clinical patients. With the cutpoint ≥10 for the two scales [32], quite few participants achieved the level of moderate to severe anxiety or depression, which means that there were few “real” patients with anxiety or depression disorders in our sample. So the validity of our model in recognizing questionnaire scores of anxiety or depression cannot be equated with the effectiveness in clinical practice, and the diagnostic performance of the model such as the sensitivity and specificity in finding patients were not yet tested. Third, the current approach was data-driven and just built the association between low-level gait features and anxiety/depression severity. For a clear description of the relationship between those intuitively visible, high-level gait features and anxiety/depression scores, further kinesiological study is necessary. Forth, although the large correlation between the predicted and questionnaire summed-item scores showed the validity of screening depression by the model, the relationship between gaits and different symptoms relevant to depression is left as an open question. The great disparity of the accuracies in detecting different symptoms implied that not all the depression-relevant symptoms could be equally reflected in gaits. As the first step, this study mainly focused on predicting the summed-item score which is the most useful in practice. But to get a better understanding on how gaits reflect certain symptoms and then the general level of depression, it still needs more indepth analysis, such as factor analysis, in future study.

Despite those limitations due to the exploratory nature of the study, it suggests the potential in future mental health services. An individual’s gait is objective and could be obtained repeatedly at any time, while requiring him/her finishing a questionnaire repeatedly and frequently is often not acceptable in practice. So our gait-based predictive model may be more suitable than questionnaires for monitoring the continuous change of anxiety/depression severity of individuals. The low volume of gait data needed and the timeliness of measurement made this method suitable for a very fast screening. In the current study, we trained and tested predictive models based on the continuous gaits data as short as 64 frames (about 2s). It means that we could possibly get enough data clips while participants naturally passing by the Kinect, and may not need to raise extra requirement of walking back and forth in practical applications. This method may also show advantages in some other situations where the use of questionnaire is restricted, such as on the population with low education level. Besides screening anxiety and depression by the predicted total scores, our classification models with high accuracy could be used to detect some certain symptoms relevant to depression, such as losing interest or pleasure, feeling down or depressed, and low energy. To reach these potentials, more future works need to continue from two aspects: First, building and testing the model with the sample of larger size and similar to the target users, such as real patients; Second, exploring the validity and availability of this method in the target scenarios, for example, the diagnostic performance of it if used as an aid to clinical judgment.

In conclusion, this study moved one step forward towards a non-intrusive, low-cost solution for real-time monitoring the metal health condition, which would be of potential value in mental health services. Our experiment demonstrated that the natural gaits could be an objective data source for measuring anxiety and depression, and the predictive models showed the effectiveness not only in recognizing the total questionnaire scores of anxiety and depression, but also in detecting some self-reported specific depressive symptoms. Though the nonpatient sample and the questionnaire-based design limited the applicability of the current model, this pilot study indicated one possible direction that is worthy of further investigation for new convenient mental health measuring methods.

## Supporting information

S1 Dataset The dataset of the study. (RAR)
